# Anterior Cruciate Ligament Reconstruction and Medial Collateral Ligament Augmentation With Pedicled Gracilis Tendon to Address Anteromedial Rotatory and Valgus Instability

**DOI:** 10.1002/atn2.70097

**Published:** 2026-05-20

**Authors:** Lukas Willinger, Julian Mehl, Sebastian Siebenlist, Armin Runer

**Affiliations:** ^1^ Department of Sports Orthopaedics TUM University Hospital, Technical University of Munich Munich Germany

## Abstract

Combined anterior cruciate ligament (ACL) and medial collateral ligament (MCL) injuries with persistent grade II valgus (Fetto/Marshall classification) or anteromedial rotatory instability pose a significant risk to ACL graft integrity if treated in isolation. This single‐stage procedure involves ACL reconstruction using a quadriceps tendon autograft combined with minimally invasive MCL augmentation using a pedicled gracilis tendon with preserved distal attachment. This gracilis‐based approach addresses both superficial MCL and deep MCL stabilization in cases with valgus laxity at 20° to 30° of knee flexion. The present article outlines graft harvest, tunnel placement, graft routing, and fixation, emphasizing isometric reconstruction and graft protection. This combined procedure is believed to restore native knee biomechanics and to reduce ACL graft failure risk.

## Graphical abstract

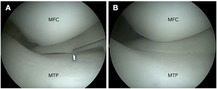

**VIDEO 1:** The video shows a step‐by‐step approach to an anteromedial medial collateral ligament (MCL) augmentation of the MCL with the ipsilateral gracilis tendon with preserved distal attachment and combined anterior cruciate ligament reconstruction with quadriceps tendon. The MCL augmentation procedure begins with identification of the gracilis tendon through an incision medial to the tibial tuberosity, followed by proximal tendon harvest using an open stripper while maintaining the native tibial insertion. A second 2‐cm incision is made over the medial femoral epicondyle to identify the anatomic femoral footprint of the superficial MCL with the help of true lateral fluoroscopy. The gracilis tendon is then routed deep to the pes anserinus fascia toward the femoral site. The free graft limb is then secured distally at the tibial superficial MCL midpoint. A 6‐mm femoral tunnel is drilled at the anatomic superficial MCL insertion, and the graft is tensioned at 20° to 30° of flexion under slight varus stress before fixation with a 6 × 20 mm biocomposite interference screw (Arthrex, Naples, FL). The graft is rerouted anteromedial and fixed with an all‐suture anchor at the anteromedial proximal tibia reproducing both superficial and deep MCL function. Final assessment shows restoration of valgus and anteromedial stability with full range of motion. Video content can be viewed at https://doi.org/10.1002/atn2.70097.

Combined anterior cruciate ligament (ACL) and medial collateral ligament (MCL) injuries represent a complex challenge in knee surgery, particularly when medial‐sided instability persists beyond the acute phase. Although low‐grade MCL injuries (grade I‐II, Fetto/Marshall classification^1^) can often be treated nonoperatively with favorable outcomes, persistent valgus laxity or anteromedial rotatory instability (AMRI)—especially in the setting of ACL deficiency—can compromise the success of isolated ACL reconstruction.^2^, ^3^ Clinical and biomechanical studies have shown that unaddressed medial laxity leads to increased graft loading and higher risk of graft failure, particularly in pivoting athletes and patients with chronic or combined injuries.^4^, ^5^

Over the past decade, there has been significant evolution in surgical techniques for MCL reconstruction, tailored to the severity and chronicity of the injury. For higher‐grade or chronic medial instability, especially in combination with ACL rupture, surgical reconstruction of the medial complex is increasingly indicated to restore both valgus and rotational stability.^6^, ^7^

These surgical techniques differ considerably in graft selection (gracilis, semitendinosus, and allograft), anatomical targeting (isolated superficial MCL [sMCL] vs combined sMCL and deep MCL [dMCL] reconstruction), and surgical approach—from open double‐bundle reconstructions to minimally invasive graft augmentations with preserved distal insertions.^8^, ^9^, ^10^, ^11^, ^12^

The choice of reconstruction technique should be guided by the degree of medial laxity, presence of AMRI, chronicity, tissue quality, and timing of intervention. This technical note describes a minimally invasive surgical strategy that combines quadriceps tendon autograft for ACL reconstruction with an anatomic, gracilis tendon‐based MCL augmentation. The technique targets both the superficial and deep MCL components, with the aim of restoring native anteromedial stability while minimizing surgical morbidity.

## SURGICAL TECHNIQUE

### Indication and Diagnosis

Indications include MCL rupture on magnetic resonance imaging with clinical confirmation of persistent grade II° MCL laxity according to Fetto and Marshall with high‐grade valgus opening (>6 mm) in 20° to 30° flexion and/or positive AMRI tests (i.e., Lachman positive with external rotation applied, positive Slocum test in 90° knee flexion).

Clinical examination includes positive Lachman and pivot‐shift test to confirm ACL insufficiency. AMRI is suspected with the Lachman test increased with additional external rotation and a positive Slocum test in 90° knee flexion. Grade II° MCL injury according to Fetto and Marshall^1^ is diagnosed by high‐grade valgus opening of the medial joint space (>6 mm) in 30° knee flexion in comparison to the contralateral knee. There is no valgus opening in full extension, confirming the integrity of the posterior medial capsule and the posterior oblique ligament.

In acute magnetic resonance imaging a rupture of the ACL and the MCL can be detected. However, in more chronic cases with delayed radiological imaging, the investigation may be incongruent to the clinical findings without signs of MCL injury.

Surgical intervention is indicated in cases of persistent anterior, anteromedial, and valgus laxity in the clinical examination. This may also occur after primary nonoperative treatment of the MCL injury by using hinged brace, when nonhealing of the MCL is evident after 6 weeks.

### Patient Positioning and Setup

The patient is placed supine under general anesthesia. A lateral post is positioned at midthigh, and a foot roll is used to allow free knee motion. A tourniquet is applied and inflated before surgery commences. Prophylactic antibiotics are administered.

### ACL Reconstruction

**FIGURE 1:**
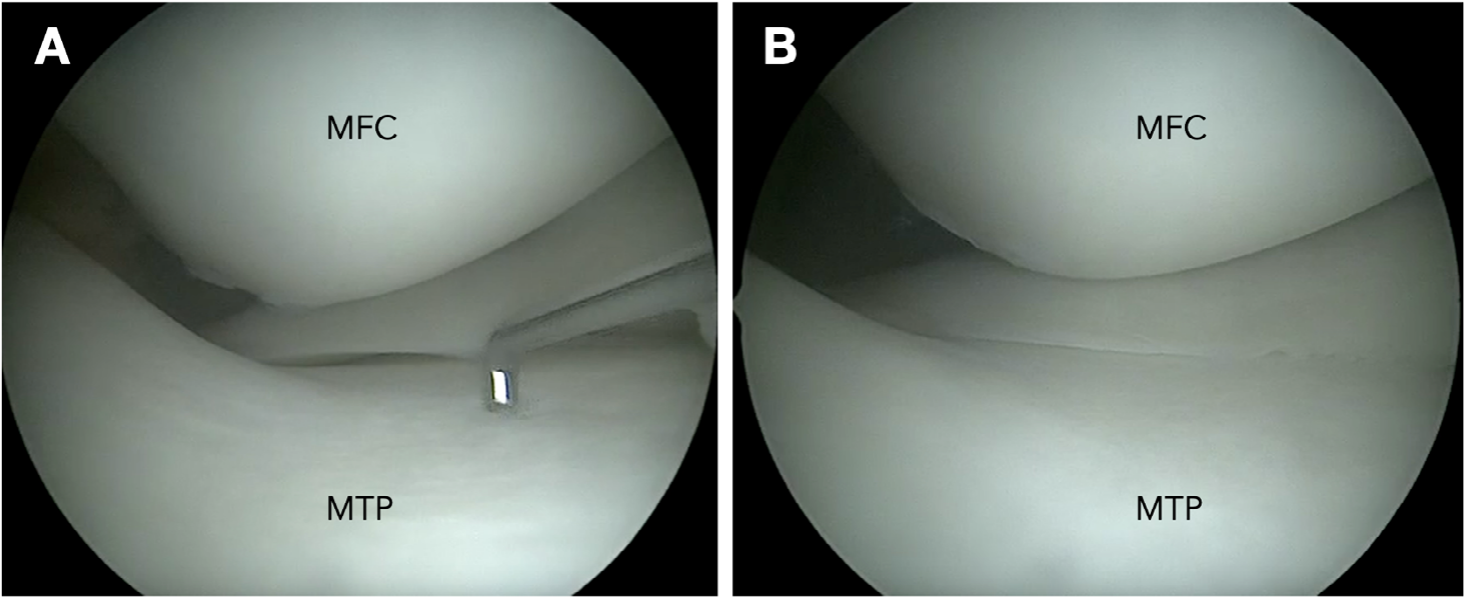
Diagnostic arthroscopy of a right knee from the anterolateral portal shows (A) a medial joint space opening between the MFC and the MTP of about 10 mm measured with a 3‐mm probe and (B) an anterior subluxation of the posterior horn of the medial meniscus on anterior draw force in 30° flexion confirming high‐grade medial laxity and anteromedial rotatory instability. (MFC, medial femoral condyle; MTP, medial tibia plateau.)

Standard anterolateral and anteromedial portals are established. Diagnostic arthroscopy is performed to confirm the ACL rupture and evaluate concomitant intra‐articular injuries. This includes also the evaluation of anteromedial and medial instability, measured by the medial joint space opening, a positive drive‐through sign and anterior subluxation of the posterior horn of the medial meniscus below the femoral condyle (Figure 1).

A quadriceps tendon autograft measuring 8 to 9 mm in diameter is harvested via a minimally invasive suprapatellar incision without a bone block. Residual ACL fibers are debrided, preserving the native tibial stump when possible to support graft healing and proprioception. The femoral tunnel is drilled through an accessory medial portal using a transportal technique, targeting the native anteromedial bundle footprint at approximately the 10:30 o'clock position. The tibial tunnel is created using an ACL guide set to approximately 60°, aiming for the anatomic tibial footprint posterior to the anterior horn of the lateral meniscus.

Graft passage is performed under direct arthroscopic visualization. Femoral fixation is achieved using suspensory cortical fixation (e.g., TightRope, Arthrex, Naples, FL or EndoButton, Smith & Nephew, Andover, MA), whereas tibial fixation is performed using an interference screw, ensuring proper tensioning at 20° to 30° of flexion. Final assessment includes probing for stability and ensuring full extension and flexion are achievable without graft impingement.

### MCL Augmentation With Gracilis Tenodesis

Through a 3‐cm longitudinal incision approximately 2 cm medial to the tibial tuberosity, the gracilis tendon is identified and harvested proximally using an open tendon stripper, with the distal insertion left in situ. This approach preserves native tibial attachment and vascular supply, potentially enhancing graft incorporation and minimizing donor‐site morbidity.^11^, ^12^

**FIGURE 2:**
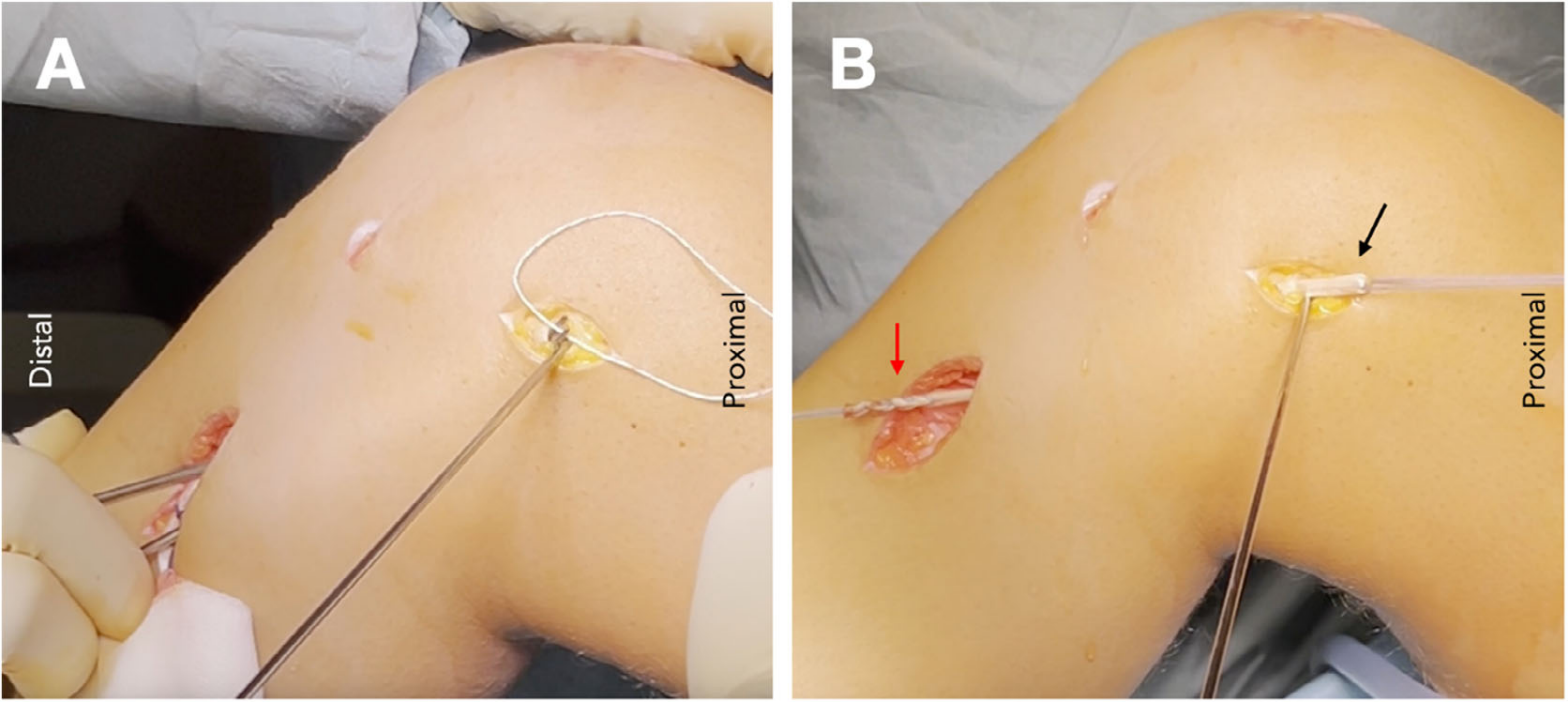
View of a right knee from the medial side. (A) A passing suture is pulled back underneath the sartorius fascia by the use of a clamp. (B) The gracilis tendon graft is shuttled proximally to the femoral attachment (black arrow) of the superficial medial collateral ligament at the medial femoral epicondyle (drill wire). The proximal free end is kept distally at the pes anserinus (red arrow).

A second 2‐cm incision is made over the femoral attachment of the sMCL, over the medial epicondyle.^13^, ^14^ The anatomic femoral MCL attachment is identified by using radiolucency^13^ and a drill pin is inserted through the femur. A gracilis graft loop is then passed deep to the sartorius fascia and superficial to the sMCL, staying extra‐articular, toward the femoral tunnel site (Video 1, Figure 2).

**FIGURE 3:**
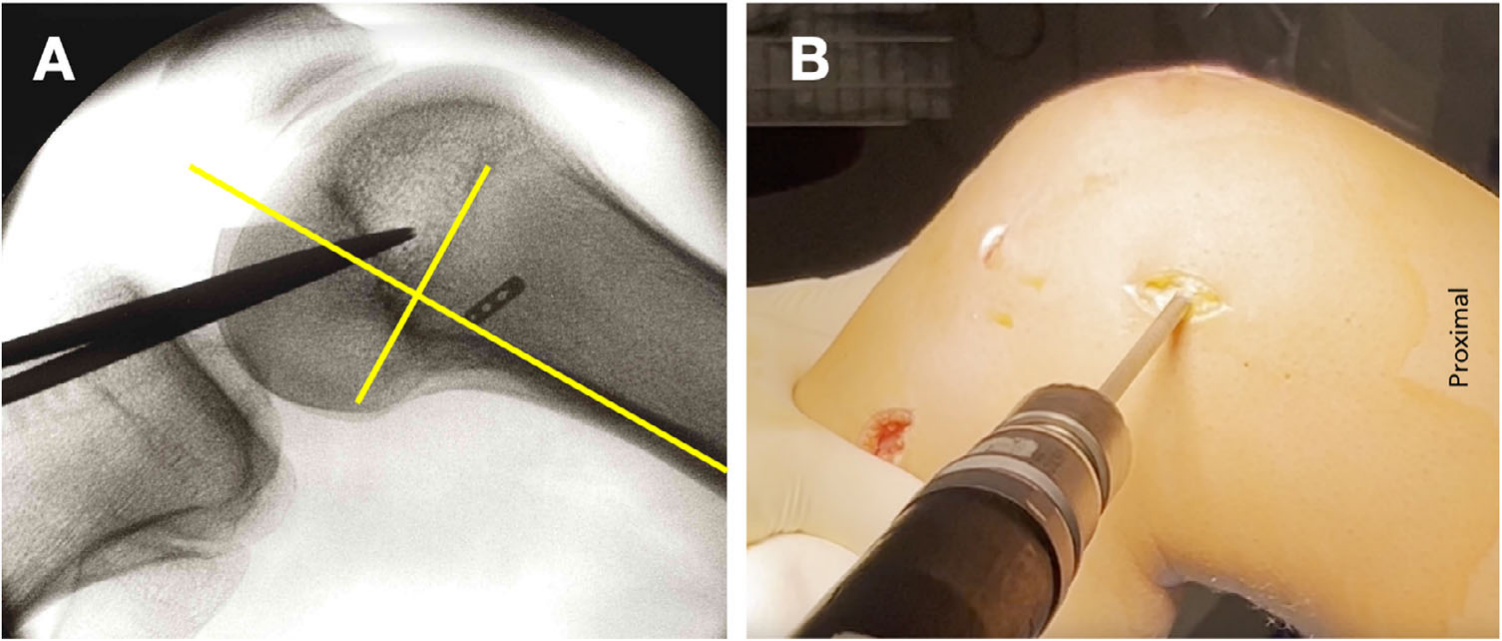
View of a right knee from the medial side. A 6‐mm femoral tunnel is drilled at the footprint of the superficial medial collateral ligament (B) under fluoroscopy guidance (A) in true lateral views. The femoral entry point is based on the anatomical and radiological description according to Athwal et al.^13^

The proximal tendon is kept distally for the following fixation. Isometry is assessed by cycling the knee through flexion‐extension to confirm minimal length change between 0° and 90°.^14^, ^15^ A 6‐mm femoral tunnel is drilled at the sMCL footprint (Figure 3).

**FIGURE 4:**
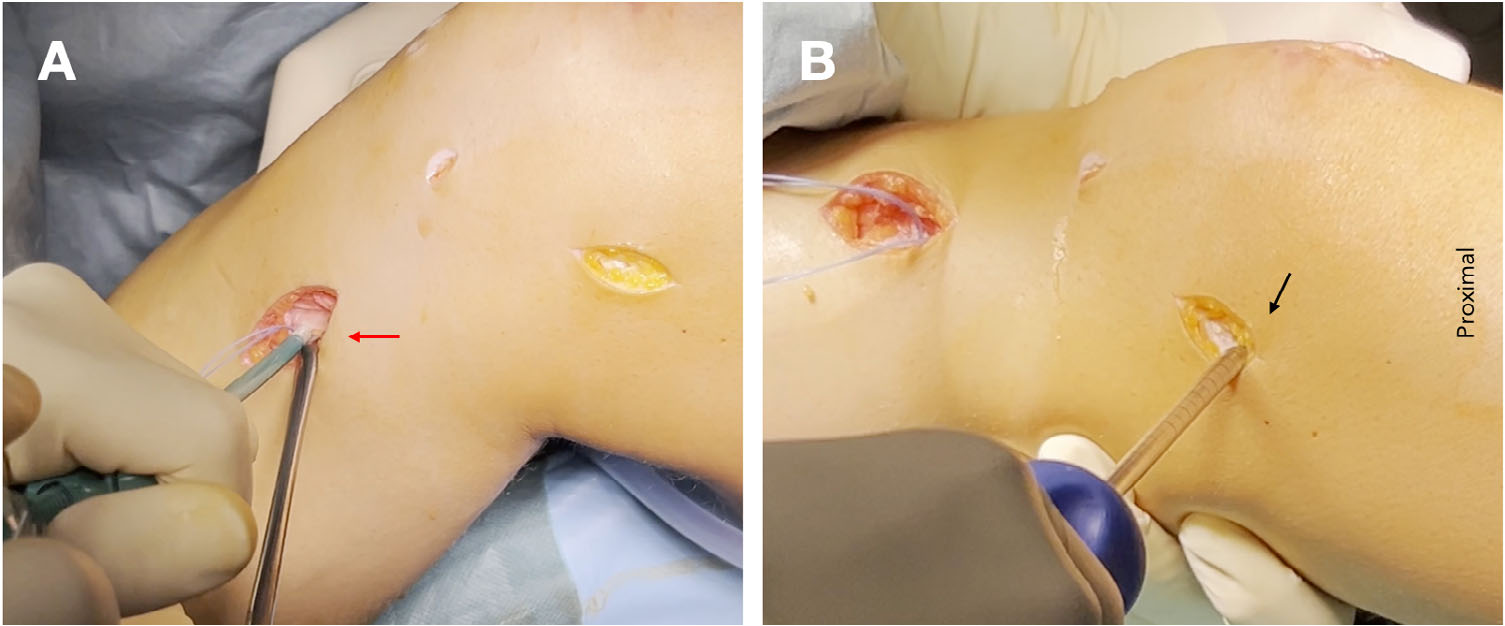
View of a right knee from the medial side. (A) The gracilis graft is first fixed at the midpoint of the distal sMCL attachment site (red arrow) with a 4.75‐mm PEEK interference screw (Arthrex, Naples, FL). (B) After tensioning, the graft is femorally fixed (black arrow) by a 6 × 20 mm biocomposite interference screw (Arthrex, Naples, FL). (sMCL, superficial medial collateral ligament.)

The graft loop is passed laterally into the tunnel. First, the free proximal limb of the graft is fixed at the midpoint of the distal sMCL attachment site approximately 6 cm distal to the joint line with a 4.75‐mm PEEK interference screw (e.g., SwiveLock, Arthrex, Naples, FL, Figure 4A). The graft is then tensioned femoral by the use of the passing suture laterally at 20° to 30° of knee flexion under slight varus stress and secured with a 6 × 20 mm biocomposite interference screw (Arthrex, Naples, FL, Figure 4B).

An all‐suture (e.g., Knee FiberTak Anchor, Artrhrex, Naples, FL) anchor is inserted 10 mm below the joint line into the anteromedial tibia. Nonabsorbable sutures are then used to reroute the anterior bundle of the graft more anteriorly and fixed in onlay technique. The oblique course of the anterior limb should better restore anteromedial stability.

**FIGURE 5:**
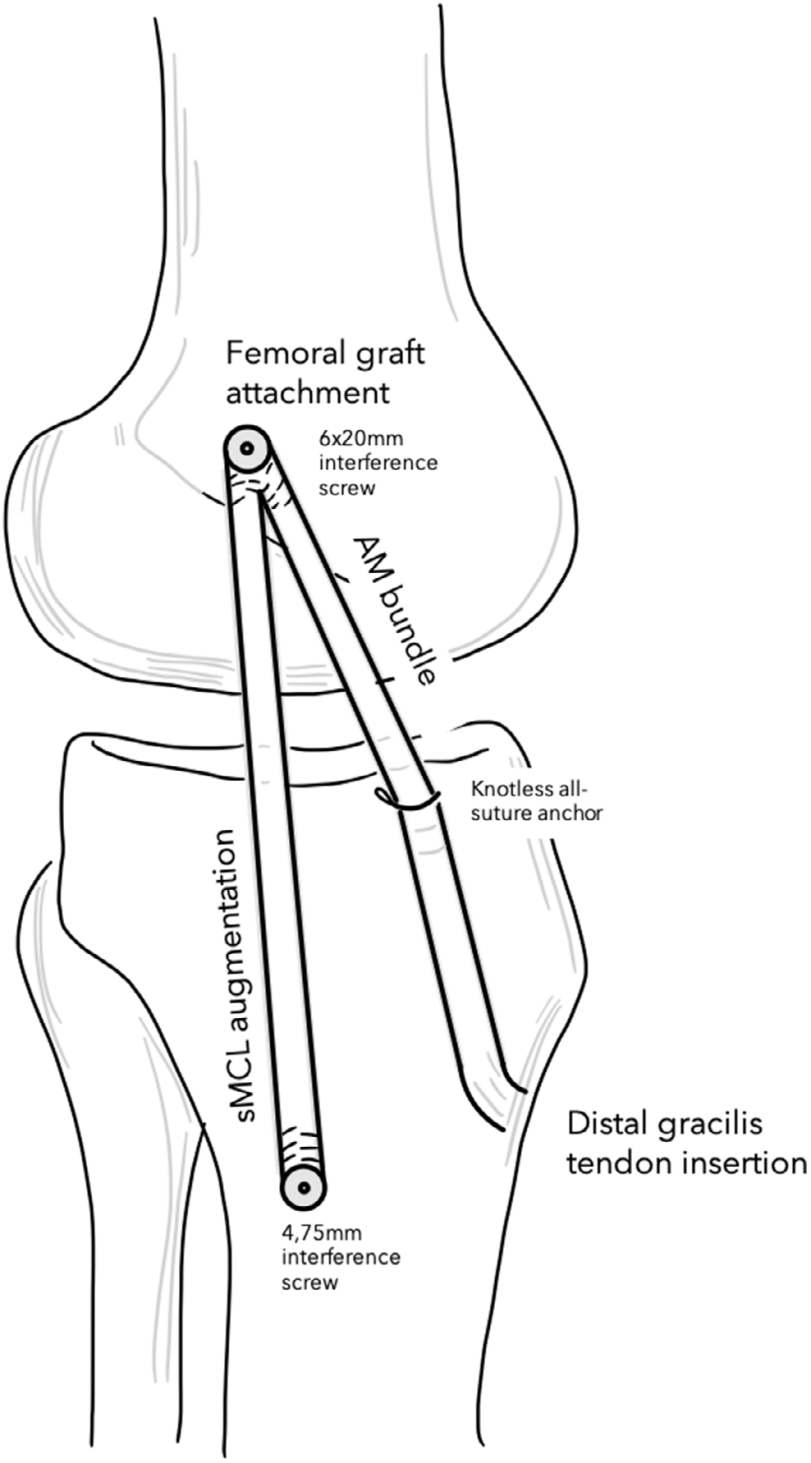
Schematic drawing of the medial collateral ligament augmentation with a pedicled gracilis tendon graft. (AM, anteromedial; sMCL, superficial medial collateral ligament.)

Hereby, both superficial and deep MCL functions are replicated, and anteromedial knee stability is restored (Figure 5).

### Closure

Skin closure is completed in layers with closure of the sartorius fascia distally. A sterile compressive dressing is applied, and a hinged knee brace is fitted.

### Postoperative Rehabilitation

Postoperatively, weight‐bearing is allowed as tolerated with the brace locked in extension for 6 weeks. Range of motion is gradually advanced:

• Weeks 1 to 2: Extension/Flexion: 10° to 90°
• Weeks 3 to 6: Extension/Flexion: 0° to 90°

Quadriceps activation is encouraged immediately. Return to low‐impact activities begins at 3 months, and pivoting sports are delayed until 9 to 12 months postoperatively.

## DISCUSSION

The presented technique combines ACL reconstruction using a quadriceps tendon autograft with a minimally invasive MCL augmentation utilizing the pedicled gracilis tendon, preserving its distal insertion (Table 1). This approach is designed to restore anteromedial knee stability in patients with combined ACL and grade II valgus laxity (according to Fetto and Marshall), while minimizing donor‐site morbidity and surgical invasiveness. Conservative treatment of MCL injuries in combination with ACL reconstruction leads to a higher failure rate compared with early surgical management.^16^, ^17^

**TABLE 1:** Pearls and Pitfalls of the Described Surgical Technique

Pearls	Pitfalls
Minimal‐invasive augmentation of the superficial and deep medial collateral ligament	Inaccurate placement of the drill tunnels would lead to premature loosening or overtensioning of the graft
Preserved distal bony attachment of the tendon (blood supply and fixation strength)	Exact use of radiolucency and isometry testing is advised for correct tunnel placement

One of the key advantages of this technique is its anatomic and biomechanically informed approach to MCL augmentation.^6^, ^7^ By preserving the distal gracilis attachment and routing the tendon anatomically between the femoral sMCL footprint and midportion of the tibial MCL, both superficial and deep MCL functions are addressed.^6^, ^7^ Fixation in slight knee flexion with varus tension reproduces the native valgus restraint behavior of the medial complex. Moreover, this method avoids a tibial tunnel, reducing the risk of tunnel convergence in multiligament surgery and preserving bone stock (Table 2).

**TABLE 2:** Advantages and Disadvantages of the Described Surgical Technique

Advantages	Disadvantages
Restore medial and anteromedial knee stability	Too weak to treat grade III° medial knee instability (Fetto/Marshall classification)
Augment the native sMCL and dMCL	Use gracilis tendon as active stabilizer of the medial side
Reduces graft stress of the ACL	
Preserves the stronger semitendinosus tendon as active medial stabilizer	

ACL, anterior cruciate ligament; dMCL, deep medial collateral ligament; sMCL, superficial medial collateral ligament.

Compared with traditional open reconstructions, this minimally invasive technique requires smaller incisions, limits soft tissue dissection, and reduces postoperative morbidity—advantages particularly relevant in athletes and active patients. Additionally, the use of quadriceps tendon for ACL reconstruction offers robust graft diameter and favorable biomechanical properties with a low rate of donor site complications.

Alternative techniques include the gracilis tenodesis method described by Wierer et al.,^12^ which also preserves the tibial insertion and reconstructs both the sMCL and dMCL in a Y‐shaped construct. Although conceptually similar to the approach described in this manuscript, it does not reroute the anterior graft to the anteromedial tibia, making it more oblique to mimic more the course of the anteromedial fibers of the dMCL (Table 3).

**TABLE 3:** List of Special Instruments Used During the Procedure

Instrument	Purpose
Drill wire and 6‐mm drill	Create femoral attachment
Open tendon stripper	Strip gracilis tendon with preserved distal attachment
SwiveLock and interference screw	Graft fixation

Another well‐established option is the semitendinosus tenodesis technique by Lind and Kittl,^11^ which also uses the native insertion to provide a tensioned, dynamic reconstruction of the medial side. Their construct provides robust control of AMRI and has been validated in biomechanical studies.^6^ Augmentation of the MCL also reduces the force on the ACL graft.^18^ However, in patients where the semitendinosus tendon may already be used or compromised (e.g., prior hamstring harvest), the gracilis remains a reliable and often underutilized alternative. In addition, using the gracilis tendon preserves the stronger semitendinosus tendon for active medial stabilization.

A critical component of successful MCL reconstruction is the correct placement of femoral and tibial insertions to ensure graft isometry and appropriate length change behavior during knee motion. Detailed analyses of the native sMCL and dMCL insertion sites, emphasizing that the femoral sMCL insertion lies oval shaped just at the medial epicondyle, while the tibial insertion extends from 6 to 10 cm distal to the joint line.^13^, ^14^ The work highlights that inappropriate graft placement can result in nonisometric constructs, leading to overconstraint or laxity throughout the range of motion.

The present technique respects these anatomical principles, ensuring graft fixation in a position that mirrors native ligament function and preserves physiologic joint kinematics.

Limitations of the described technique include the technical demand of achieving accurate femoral tunnel placement and isometry through small incisions. Future biomechanical and comparative studies with patient‐reported outcome measures and failure rates will be essential to validate the efficacy of this approach in various populations.

## DECLARATION OF GENERATIVE AI AND AI‐ASSISTED TECHNOLOGIES IN THE WRITING PROCESS

During the preparation of this work, the authors used ChatGPT (ChatGPT‐4 Turbo, OpenAI) in order to assist with language refinement and structuring of the manuscript. After using this tool, the authors reviewed and edited the content as needed and take full responsibility for the content of the publication.

## DISCLOSURES

The authors (L.W., J.M., S.S., A.R.) declare that they have no known competing financial interests or personal relationships that could have appeared to influence the work reported in this article.

## FUNDING

Open Access funding enabled and organized by Projekt DEAL.
